# Supplementary material for: Variation in Lipid Species Profiles among Leukemic Cells Significantly Impacts Their Sensitivity to the Drug Targeting of Lipid Metabolism and the Prognosis of AML Patients
Source: Int J Mol Sci. 2023 Mar 22;24(6):5988. doi: 10.3390/ijms24065988 (PMC10054724; doi:10.3390/ijms24065988)
Supplement: Supplementary file 1 [file ijms-24-05988-s001.zip › ijms-2084396-supplementary.pdf]

Supplemental Table S1. Patient's characteristics at diagnosis of *de novo* AML (n = 39)

**Median age**	58 [24 ; 88]
**Sex ratio (M/W)**	2
**Leukocytosis**	**n (%)**
< 4 10^3/µL	8 (20)
4-30 10^3/µL	17 (44)
30-100 10^3/µL	11 (28)
> 100 10^3/µL	3 (8)
**AML subtype (FAB classification)**	**n (%)**
M0	2 (5)
M1	9 (23)
M2	18 (46)
M4	6 (16)
M5	4 (10)
**Bone marrow blasts**	**n (%)**
20-40%	9 (23)
40-60%	11 (28)
60-80%	9 (23)
80-100%	10 (26)
**Prognosis (ELN 2017)**	**n (%)**
Favorable	21 (54)
Adverse	18 (46)
**Molecular status**	**n (%)**
NPM1	15 (38)
FLT3-ITD	11 (28)
IDH	9 (23)
TET2	4 (10)
CEBPA	4 (10)
RUNX1	1 (3)
TP53	1 (3)
**Response to chemotherapy**	**n (%)**
Chemosensitive	20 (51)
Chemoresistant	6 (16)
Non available	13 (33)
